# *In vivo* Overexpression of Electrogenic Sodium/Bicarbonate Cotransporter (NBCe1) by AAV9 Modifies the Cardiac Action Potential and the QT Interval in Mice

**DOI:** 10.3389/fcvm.2022.862118

**Published:** 2022-04-25

**Authors:** Romina A. Di Mattía, Leandro A. Díaz Zegarra, Carlos A. Valverde, Paula G. Blanco, Carolina Jaquenod De Giusti, Enrique L. Portiansky, Ernesto A. Aiello, Alejandro Orlowski

**Affiliations:** ^1^Centro de Investigaciones Cardiovasculares “Dr. Horacio E. Cingolani, ” Facultad de Ciencias Médicas, Universidad Nacional de La Plata-CONICET, La Plata, Argentina; ^2^Centro de Fisiología Reproductiva y Métodos Complementarios de Diagnóstico, Facultad de Ciencias Veterinarias, Universidad Nacional de La Plata-CONICET, La Plata, Argentina; ^3^Laboratorio de Análisis de Imágenes, Facultad de Ciencias Veterinarias, Universidad Nacional de La Plata-CONICET, La Plata, Argentina

**Keywords:** NBCe1, adeno-associated virus (AAV), ECG, action potential, mice

## Abstract

Cardiac cells depend on specific sarcolemmal ion transporters to assure the correct intracellular pH regulation. The sodium/bicarbonate cotransporter (NBC) is one of the major alkalinizing mechanisms. In the heart two different NBC isoforms have been described: the electroneutral NBCn1 (1Na^+^:1${\text{HCO}}_{3}^{-}$) and the electrogenic NBCe1 (1Na^+^:2${\text{HCO}}_{3}^{-}$). NBCe1 generates an anionic repolarizing current that modulates the action potential duration (APD). In addition to regulating the pH, the NBC is a source of sodium influx. It has been postulated that NBC could play a role in the development of hypertrophy. The aim of this research was to study the contribution of NBCe1 in heart electrophysiology and in the development of heart hypertrophy in an *in vivo* mouse model with overexpression of NBCe1. Heart NBCe1 overexpression was achieved by a recombinant cardiotropic adeno-associated virus (AAV9) and was evidenced by western-blot and qPCR. AAV9-mCherry was used as a transduction control. NBCe1 overexpression fails to increase heart growth. Patch clamp and electrocardiogram were performed. We observed a reduction on both, ventricular myocytes APD and electrocardiogram QT interval corrected by cardiac rate, emphasizing for the first time NBCe1 relevance for the electrical activity of the heart.

## Introduction

Intracellular pH (pH_i_) maintenance is required for any physiological process. Specifically, in cardiomyocytes is crucial for the correct function of many key features, such as electrical activity and contractility, which cannot be guaranteed under acidic conditions. To prevent acidosis, cardiac cells rely on two sarcolemmal acid extruders: Na^+^/H^+^ exchanger or NHE1 and the Na^+^/${\text{HCO}}_{3}^{-}$ cotransporter or NBC, which drives the co-influx of an ion of Na^+^ and one or two molecules of ${\text{HCO}}_{3}^{-}$ (1). In the heart, ${\text{HCO}}_{3}^{-}$ flux is responsible for about 40–50% of the pH_i_ regulation (2).

Two NBC isoforms have been described in cardiomyocytes: electrogenic NBC or NBCe1 and the electroneutral NBC or NBCn1. NBCe1 catalyzes the co-influx of two ${\text{HCO}}_{3}^{-}$ per each Na^+^, therefore, in each transport cycle, a negative charge enters the cell, generating an anionic current denominated I_NBC_. Our laboratory was the first to demonstrate in isolated ventricular myocytes of rat (3) and cat (4) the existence of I_NBC_ and its contribution to the shape and duration of the cardiac action potential. Similar results were obtained by other laboratories in isolated ventricular myocytes of guinea pigs and rabbits (5). However, so far, the contribution of I_NBC_ has been only shown in *in vitro* models.

Several studies have associated the NBC activity with the hypertrophic heart growth (6–8). In addition to regulating the intracellular pH, NBC promotes Na^+^ influx. It has been shown that increased intracellular Na^+^ can activate pro-hypertrophic pathways due to increased intracellular Ca^2+^ by sodium/calcium exchanger (NCX) (9, 10). However, the contribution of NBCe1 on cardiac hypertrophy development is still not clear.

The principal aim of the present research is to study the contribution of NBCe1 in heart electrophysiology and in the development of heart hypertrophy in an *in vivo* mouse model with overexpression of NBCe1. Herein, through administration of a recombinant cardiotropic adeno-associated virus (rAAV9) we attempted to describe electrical cardiac features based on patch clamp and electrocardiogram (ECG) studies in these rodents. Moreover, we studied the contribution of overexpressing NBCe1 in hypertrophy heart development.

## Materials and Methods

All procedures followed during this investigation conform to the Guide for the Care and Use of Laboratory Animals published by the US National Institutes of Health (NIH Publication No. 85–23, revised 1996) and the experimental protocol was approved by the Animal Welfare Committee of La Plata School of Medicine.

### Myocytes Isolation

Ventricular myocytes were isolated according to the technique previously described (11), using 4-month-old male c57bl/6 mice.

### AAV9-NBCe1 and AAV9-MCherry Production

Recombinant AAV9-NBCe1 or AAV9-mCherry was produced essentially as previously described (12). HEK293T/17 (CRL-11268, ATCC) cells were cultured with DMEM supplemented with 10% fetal bovine serum and penicillin/streptomycin at 37°C and 5% CO_2_. Once 90% confluence was reached, transfection was performed by adding *cis*-plasmid (pNBCe1 and pmCherry were obtain from VectorBuilder^®^), pDG9 (gift from Dr. Roger Hajjar), and PEI-max (*Polysciences*) solution (pH 7). The solution was vortexed for 10 s and incubated at room temperature for 15 min. The transfection mix was then added to pre-warmed DMEM containing 2% FBS. The medium from the plate was removed and replaced with the transfection mixture. After 3 days of growth, the cells were harvested and the supernatant was kept for downstream processing. The cell pellet was resuspended in 5 mL of lysis buffer (150 mM of NaCl, 50 mM of Tris hydrochloride, pH 8.5, and 2 mM of MgCl_2_), and freeze-thawed three times at −80°C and 37°C with brief vortexing after each thaw cycle. Non-encapsidated DNA and contaminating RNA was then digested by adding 2 μL (10 IU/μL) of Pierce Universal Nuclease (*Thermo Fisher Scientific, Waltham, USA*) followed by incubation for 30 min at room temperature. The crude cell lysate was centrifuged to pellet debris, and the supernatant was reserved for iodixanol (*Sigma Aldrich, St. Louis, USA*) gradient ultracentrifugation. Virus from the cell culture supernatant was precipitated by the addition of 31.3 g of ammonium sulfate per 100 mL of supernatant followed by incubation on ice for at least 30 min. The precipitate was pelleted by centrifugation, resuspended in 5 mL of lysis buffer, and then combined with the cell pellet supernatant for iodixanol gradient ultracentrifugation. Samples for ultracentrifugation were prepared in polypropylene Optiseal tubes (*Beckman Coulter, Brea, USA*). Viral lysates were loaded on top of discontinuous iodixanol gradients composed of 4 mL of 60% iodixanol, 4 mL of 40%, 4.9 mL of 25%, and 7.3 mL of 17% (with 1 M sodium chloride). The gradients were centrifuged at 350,333 × g (avg) for 60 min at 18°C in a Beckman type 70 Ti fixed angle rotor. Fractions (1.25 mL) were collected from the bottom of the tube and kept for virus titration. Peak fractions were dialyzed in lactated Ringer's solution (Baxter International), filtered through a 0.22 μm pore filter (*Merck Millipore, Burlington, USA*), and stored at −80°C. Mice anaesthetised with an inhalant anesthetic and an ophthalmic anesthetic (0.5% propacaine) were retro-orbital injected with AAV with a 27.5-gauge needle.

### Western Blot Analysis

Ventricle samples were lysed in a RIPA buffer with protease and phosphatase inhibitors cocktail. After a brief centrifugation, the supernatant was kept and protein content was quantified with Bio-Rad Protein Assay through Bradford method. Protein samples were separated by electrophoresis on 8% sodium dodecyl sulfate-polyacrylamide gel (SDS-PAGE) and transferred to PVDF membranes. Membranes were then blocked with non-fat-dry milk and incubated overnight with anti-loop 3 [anti-NBCe1 produced in our laboratory (4)] and anti-GAPDH (1:1,000). Peroxidase conjugated anti-rabbit (sc-2004; *Santa Cruz Biotechnology, Dallas, USA*, 1:10,000) or m-IgGκ BP (sc-516102; *Santa Cruz Biotechnology, Dallas, USA*, 1:10,000) were used as secondary antibodies and bands were visualized using the ECL-Plus chemiluminescence detection system (Amersham). Blots were visualized using a Chemidoc Image Station (*Bio-Rad, Hercules, USA*) and quantified by densitometry analysis (*Image J Fiji*).

### Real Time PCR

RNA was extracted from hearts using TRIzol reagent (*Life Technologies, Carlsbad, USA*). cDNA was generated by reverse transcriptase reaction using M-MLV RT (*Promega, Madison, USA*). Real time quantitative PCR was performed on cDNA using the IQ SYBR green Super Mix (Bio-Rad, Hercules, CA) and iCycler iQ (*Bio-Rad*). The following primers were used: NBCe1 Gene Sense: GGGAGGTTGACTTCTTGGA, Antisense: CCCTTTGGACCTAAGAGAAT; GAPDH Gene Sense: CATGGCCTTCCGTGTTCCTA, and Antisense: TGCTTCACCACCTTCTTGATG. Relative abundance of RNA was calculated by the ΔΔCt method. Primers were designed using Primer-Blast (NCBI, NIH). All primers were 90–110% efficient, as assessed by standard curve, and all displayed only one dissociation peak.

### Echocardiographic Study

Mice were manipulated daily 1 week before the echocardiographic study for their habituation. Conscious mice held by the operator were monitored echocardiographically at the start and end of the treatment by 2-dimensional M-mode echocardiography with a 15-MHz transducer. Measurements were performed according to the method proposed by the American Society of Echocardiography. Left ventricular mass was calculated using Devereux's equation modified for rodents and normalized with the weight of the animal to obtain left ventricular mass index (LVMI) (1).

### Cross Sectional Area Measurement

Ventricular tissue was fixed in buffered 10% formaldehyde and paraffin embedded. LV sections (5 μm thick) at the equator were stained with hematoxylin eosin for determining cardiomyocyte cross-sectional area (CSA). To assess CSA, only round cells with visible round nucleus were considered, and 50 cells were counted in at least 10 images obtained from each left ventricle. Each cell was individually traced and its cross sectional area directly determined. All the stained sections were observed under the microscope (Olympus BX-53, Tokyo, Japan) and the images were captured using a digital video camera (Olympus DP-71). Images were digitized and processed by a computer software (*Image-Pro Plus*). The investigator responsible for the morphological analysis was blinded as to each experimental group.

### Basal Intracellular pH Measurement

pH_i_ was measured in single mice ventricular myocytes with an epi-fluorescence system (Ion Optix). Myocytes were incubated at room temperature for 10 min with 10 μM BCECF-AM followed by 30 min washout. Dye-loaded cells were placed in a chamber on the stage of an inverted microscope (Nikon TE 2000-U) and continuously superfused with a solution containing (mM) 5 KCl, 118 NaCl, 1.2 MgSO_4_, 0.8 Cl_2_Mg, 1.35 Cl_2_Ca, 10 glucose, 20 NaHCO_3_, and pH 7.4 after continuous bubbling with 5% CO_2_ and 95% O_2_. The myocytes were stimulated *via* two-platinum electrodes on either side of the bath at 0.5 Hz. Dual excitation (440 and 495 nm) was provided by a 75-watt Xenon arc lamp and transmitted to the myocytes. Emitted fluorescence was collected with a photomultiplier tube equipped with a band-pass filter centered at 535 nm. The 495-to-440 nm fluorescence ratio was digitized at 10 kHz (ION WIZARD fluorescence analysis software).

### Patch-Clamp Recordings

Action potentials (APs) were recorded with the Nystatin perforated Whole-Cell configuration of the Patch Clamp technique using current-clamp recording. Axopatch 200B amplifier and analog-to-digital converter Digidata 1322A (*Molecular Devices*) were used to acquire APs recorded with pClamp 9.2 Software (*Molecular Device*). Borosilicate patch pipettes were pulled with P-97 puller (*Sutter Instruments, Novato, USA*) to a final resistance of 2–3.5 MΩ. The pipette was filled with (in mmol l^−1^): 125 K-Gluconate, 10 KCl, 8 Na-Gluconate, 1 MgCl_2_, 10 HEPES and 0.30 mg ml-1 of Nystatin (pH 7.2 with KOH, final concentration of K^+^: 140 mmol l^−1^). The ${\text{HCO}}_{3}^{-}$ solution contained (in mmol l-1): 120 NaCl, 5 KCl, 2 CaCl_2_, 1 MgCl_2_, 20 NaHCO_3_, 5 Glucose and 13 Choline-Cl (pH 7.4 with 95% O_2_-5% CO_2_). APs were triggered by 2 ms squared pulses of depolarizing current at 1 Hz. All data recorded was compensated with the Junction Potential of −14.7 mV in the ${\text{HCO}}_{3}^{-}$ solution (Junction Potential was calculated with the Junction Potential Tools of pClamp Clampex 10.3 Software). The Patch Clamp data were processed with ClampFit 10.3 (*Molecular Device*) and analyzed in GraphPad Prism 6. All experiments were performed at room temperature (22–25°C).

### Electrocardiographic Study

Surface ECGs in c57BL/6 mice were acquired at 40 KHz sampling rate using standard ECG electrodes (lead I) for the PowerLab 4ST data acquisition system (*Adintruments, Sidney, Australia*) as previously described (13). Mice were manipulated daily 1 week before the ECG assessment for their habituation. On the day of the experiment, they were transiently sedated with a volatile anesthetic (isoflurane) in order to manipulate the animals. Recordings were performed for 2 min by placing the electrodes on the chest of the conscious tethered mouse, and after verifying a stable heart rate. For measuring QT interval, ~50 ECG traces were averaged. QT corrected (QTc) values were obtained from the Mitchel formula (Bazett formula normalized to the mean cycle length of the mice: $QTc=\frac{QT}{\sqrt{\frac{RR}{100}}}$ (14), wherein RR is the averaged period between the R waves of two consecutive traces).

### Statistics

Statistical analyses were performed using version 8 of Prism (GraphPad) and R version 4.1.2. All the data sets were analyzed with the Shapiro–Wilk normality test. Therefore, group-to-group comparisons were performed using *t*-student (normal distribution) or Mann–Whitney test (not normal distribution). Data were expressed in the text and figures as means ± S.E.M except Figure 3 that were expressed as box and whisker plot. A value of *P* < 0.05 was considered statistically significant (two-tailed test). The investigators responsible for each experiment were blinded as to each experimental group.

## Results

### *In vivo* Overexpression of NBCe1

**Figure 1:**
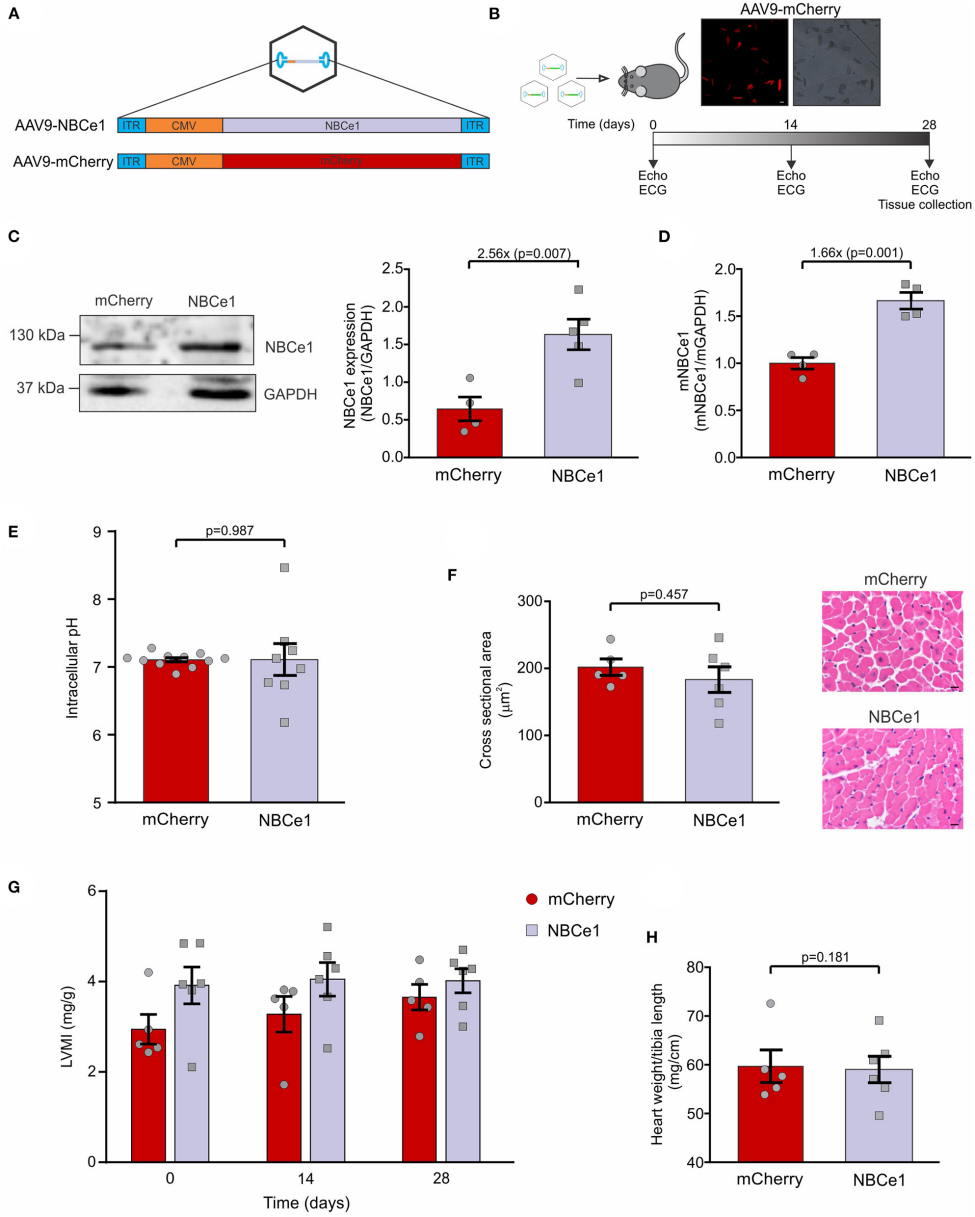
*In vivo* overexpression of NBCe1 in mice. **(A)** Diagram of packaging vector containing AAV2-ITRs and the cytomegalovirus (CMV)-driven NBCe1 or mCherry transgene. These transgenes were packaged within cardiotropic adeno-associated virus 9 (AAV9). **(B)** Scheme of the experimental design. Echocardiography and ECG recordings were obtained before and after 14 and 28 days of AAV9 tail injection. On day 28 mice were sacrificed. (Top) Fluorescent microscope images of isolated cardiomyocytes showing mCherry transduction. White scale bar corresponds to: 25μm. **(C)** NBCe1 protein expression was determined by western-blot. A significant increase of NBCe1 was found after 28 days of AAV9-NBCe1 injection. **(D)** Similar results were obtained when mRNA levels were measured by qPCR. **(E)** Average and individual values of intracellular pH in isolated adult ventricular myocytes. **(F)** (Right) Representative cross-sections area (CSA) of cardiomyocytes stained with hematoxylin-eosin technique. Black scale bar corresponds to: 10μm. (left) Quantitative analysis of cardiac myocytes CSA, non-significant differences were found 28 days post injection of AAV9-mCherry or AAV9-NBCe1. **(G)** Left ventricular mass index (LVMI) of AAV9-mCherry and AAV9-NBCe1 injected mice. Echocardiographic follow-up of the left ventricular mass index (LVMI) of the mice 14 and 28 days after injection with AAV9-mCherry and AAV9-NBCe1. **(H)** Non-evidence of hypertrophy was found when the heart weight to body weight ratio were measured. Statistical analysis was done by *t*-student test after Shapiro–Wilk normality test, *p* < 0.05 was considered significantly different.

NBCe1 overexpression virus (AAV9-NBCe1) along with its control which overexpress mCherry red fluorescent protein (AAV9-mCherry) were administered in 3-months old male mice *via* retro orbital injection (Figure 1A). Both echocardiography and electrocardiogram studies were performed before and during treatment. After 28 days, heart samples were collected and cardiomyocyte isolation was achieved. Experimental scheme is presented in Figure 1B.

Mice isolated cardiomyocytes were visualized under a fluorescent microscope and, as expected, only mCherry overexpressed mice expressed red cells (Figure 1C). This allowed us to confirm high efficiency virus transduction. Additionally, NBCe1 protein expression was measured and mice injected with AAV9-NBCe1 showed significant NBCe1 overexpression compared to control mice injected with AAV9-mCherry (AAV9-mCherry: 0.645 ± 0.15, *N* = 4; AAV9-NBCe1: 1.63 ± 0.45, *N* = 5; Figure 1D). Similar results were obtained when NBCe1 mRNA was measured by qPCR (AAV9-mCherry: 1 ± 0.06, *N* = 4; AAV9-NBCe1: 1.66 ± 0.09, *N* = 4). These results demonstrated that the overexpression of NBCe1 in the heart was achieved by a single injection of AAV9-NBCe1. In addition, the overexpression of this alkalinizing mechanism did not modify the basal intracellular pH (Figure 1E).

### NBCe1 Overexpressing Mice Did Not Exhibit Cardiac Hypertrophy

To investigate the possibility that NBCe1 overexpression could lead to cardiac hypertrophy, due to Na^+^ and Ca^2+^ overload, we conducted a series of experiments to assess heart size on both NBCe1 and mCherry overexpressed mice. Cardiomyocyte's cross-sectional area was measured on histological stained sections and none of the evaluated cells were different from each other in any of the experimental clusters (Figure 1F). Moreover, heart weight/tibia length ratio was calculated after sacrifice and no differences were found between the groups (Figure 1H). In concordance, echocardiographic studies performed before and during 28 days treatment confirmed these results: left ventricular mass index (LVMI) of NBCe1 overexpressed mice was equal to the control group (Figure 1G). Therefore, in spite of cardiac NBCe1 overexpression, we have not detected an inadequate heart growth.

### *In vivo* Chronic NBCe1 Overexpression Reduced Action Potential Duration of Cardiomyocytes

**Figure 2:**
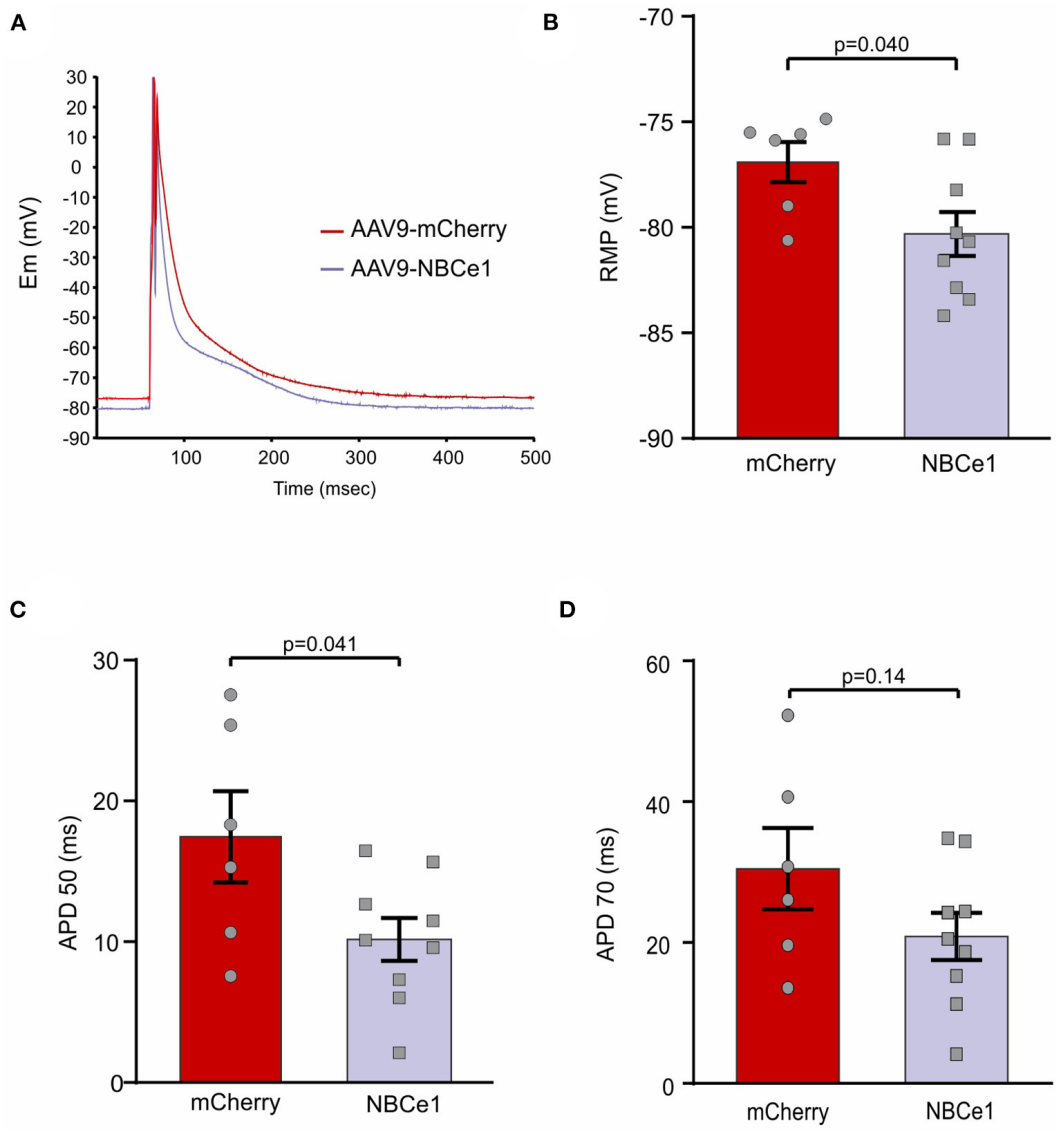
Action potential recordings. **(A)** Representative recordings of action potential (AP) in cardiomyocytes from mice after 28 days of transduction with AAV9-mCherry and AAV9-NBCe1. These representative traces of action potentials are the average of all the traces used for the electrophysiological parameters measured in this work. These traces were aligned at the peak of the action potentials. **(B)** Quantitative analysis of resting membrane potential (RMP). Mice overexpressing NBCe1 presents a significant hyperpolarization of RMP. **(C,D)** AP duration at 50% [**(C)** APD50) and 70% [**(D)** APD70] of repolarization. Statistical analysis was done by *t*-student test after Shapiro–Wilk normality test, *p* < 0.05 was considered significantly different.

Taking into account our previous findings regarding NBCe1 anionic current and its effects on action potential duration (APD) of cardiac cells, we considered the importance of studying this feature in our NBCe1 overexpression *in vivo* model. Isolated cardiomyocytes were superfused with a bicarbonate buffer and APD was measured employing patch clamp technique (perforated patch) (Figure 2A). A significant reduction in action potential duration at 50% of repolarization (APD_50_) was found in cardiomyocytes of mice previously injected with AAV9-NBCe1 compared to control AAV9-mCherry cells (Figure 2C). Although there is a clear tendency for a shorter APD_70_ in the NBCe1-overexpressed mice in comparison to control (Figure 2D), the statistical difference was not significant. In addition, APD at −50 and −70 mV were also measured and the data was included in Supplementary Figure S2. APD_−50*mV*_ and APD_−70*mV*_ were lower in NBCe1 overexpressed mice than in control. However, the difference was only statistically significant at the more depolarized value of membrane potential, like the results of APD_50_ and APD_70_. We also observed a hyperpolarization of resting membrane potential (RMP) in these NBCe1 overexpressed cells (Figure 2B). These effects were not observed in ${\text{HCO}}_{3}^{-}$ free solution (Supplementary Figure S1). These findings are consistent with the NBCe1 overexpression described above in the treated mice group.

### NBCe1 Overexpressing Mice Showed a Shortening in QTc Interval of ECG

**Figure 3:**
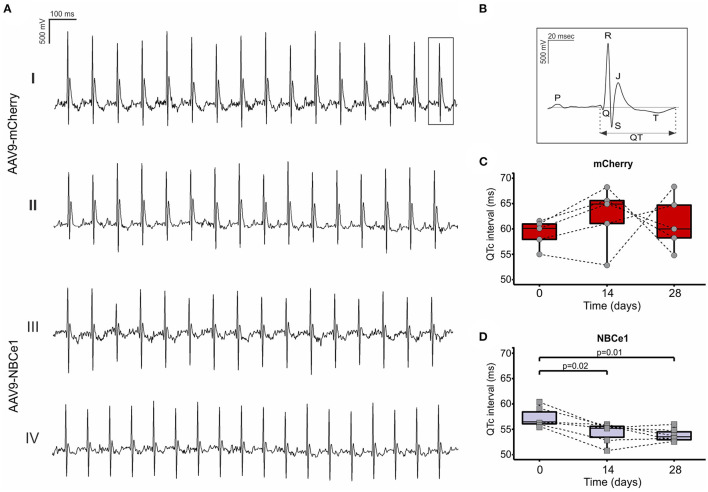
**(A)** Representative electrocardiogram (ECG) recordings of mice before (I) and 28 days after (II) AAV9-mCherry or AAV9-NBCe1 transduction (III) and (IV). **(B)** Representative surface electrocardiography (ECG) curves showing QT interval. QTc (QT interval corrected with frequency) follow-up of the mice 14 and 28 days after injection with AAV9-mCherry **(C)** and AAV9-NBCe1 **(D)**. The QTc interval was significantly shorter in AAV9-NBCe1-injected mice. Statistical analysis was done by *t*-student test after Shapiro–Wilk normality test, *p* < 0.05 was considered significantly different. Black dashed lines connect the same animals at time 0, 14, and after 28 days. Boxes correspond to upper and lower quartiles, the horizontal line represents the median, and the whiskers mark the minimum and maximum values. Statistical analysis was done by *t*-student test after Shapiro–Wilk normality test, *p* < 0.05 was considered significantly different.

Changes on electrical activity of cardiac cells are often reflected on electrocardiogram (ECG) evaluation. Particularly, QT interval corrected with cardiac frequency (QTc) provides important information about ventricular electrical activity. QTc interval durations were examined before and after 28 days of both virus injections and we found a significant reduction in mice injected with AAV9-NBCe1 compared to control (Figures 3C,D). Moreover, this QTc interval shortening was achieved after 14 days of treatment and sustained through time [QT_c_ (ms): AAV9-mCherry: 60.97 ± 0.99, *N* = 5; AAV9-NBCe1: 55.12 ± 1.09, *N* = 6).

## Discussion

The main objective of this study was to describe the contribution of the anionic repolarizing current generated by the activity of NBCe1 to the heart electrical activity. Moreover, we studied the possible effect of NBCe1 overexpression in the development of cardiac hypertrophy. Cardiac NBCe1 overexpression was achieved 28 days after a single systemic administration of adeno-associated virus serotype 9 (AAV9-NBCe1) in mice. At this time, no differences were found in the size of the heart and area of cardiomyocytes. When we studied the electrical features we found that ventricular myocytes action potential duration (APD) and electrocardiogram QT interval corrected by cardiac rate were significantly decreased compared to the control group. This is the first report of *in vivo* contribution of the NBCe1 current to cardiac action potential configuration, and the experiments reported herein suggest its physiological importance to the electrical activity of the heart.

AAV is the leading tool for *in vivo* cardiac gene transfer. However, the limitations of the size of the genome to be packaged (~4.7 Kb) makes it impossible to express large proteins (NBCe1) together with tag fluorescent proteins (15). Transduction with the control (AAV9-mCherry) yielded a transduction efficiency of more than 95% of the cardiomyocytes. In addition, AAV9-NBCe1 increases 2.56 times the expression of proteins and 1.66 times the expression of messenger RNA, which leads us to conclude that the transduction with AAV9-NBCe1 was effective in the cardiomyocytes.

The overexpression of an alkalizing mechanism, such as NBCe1, could modify the basal intracellular pH. However, no difference was found, probably due to a compensation of the acidifying mechanisms present in cardiac cardiomyocytes, mainly the anion exchanger Cl^−^/${\text{HCO}}_{3}^{-}$ (AE) and/or Slc26a6. More studies of the model will be necessary to elucidate the modification of other pH regulatory mechanisms in this model.

As NBCe1 coordinates the entrance of sodium and bicarbonate into the cell, it is feasible to think that its overexpression could increase intracellular sodium and therefore activates the reverse mode of the sodium-calcium exchanger (rNCX) leading to calcium overload, a well-known hypertrophic signal (16, 17). However, there was no evidence of ventricular mass enlargement, nor by echocardiographic imaging measurements, nor by morphometric analysis of cardiac tissue. Similar results have been previously reported in models of NBCe1 overexpression in transgenic mice (18). This could be explained in part by the stoichiometry of NBCe1 which results in less Na^+^ per ${\text{HCO}}_{3}^{-}$ when it is compared with the electroneutral NBC isoform (NBCn1). Future experiments overexpressing NBCn1 would be necessary to describe the contribution of NBC isoforms to the development of cardiac hypertrophy.

NBCe1 current has already been described by our laboratory in isolated cat and rat ventricular myocytes as a sodium and bicarbonate-dependent anionic repolarizing current that generates an APD shortening of ~25% (3, 4, 19). Additionally, the use of inhibitory (aL3) and stimulatory (aL4) polyclonal antibodies against NBCe1 has affected cardiac APD, by increasing and decreasing it, respectively (20).

In the present study we are suggesting that the electrophysiological changes observed in the AP and QTc duration are exclusively due to upregulation of I_NBC_ after overexpression of NBCe1. Although the influence of genetic manipulation on other ionic currents cannot be completely discarded, the fact that no electrophysiological changes were observed in APD or RMP in the absence of bicarbonate points to I_NBC_ as the current involved in the observed effects when the physiological buffer is present in the extracellular media.

The I_NBC_-induced APD shortening was detected when APD_50_ but not APD_70_ was measured. Since the expected I_NBC_ reversal potential is close to −95 mV, a feasible explanation for this observation is that the impact of this current on AP waveform is more important at depolarized potentials than at potentials close to RMP. On the other hand, a significant reduction of QTc was evident in the *in vivo* experiments. As the ECG recordings were measured *in vivo*, the physiological heart rate and temperature would have helped to detect the late repolarizing impact of I_NBC_ on the duration of the QT interval.

Data shown herein provides evidence of the active participation of electrogenic sodium-bicarbonate cotransporter NBCe1 through its repolarizing current (I_NBC_) upon duration of the cardiac action potential and the QT interval in the ECG, demonstrating for the first time its relevance in an *in vivo* mice model and thus becoming an actor to consider in future electrophysiological studies.

## Data Availability Statement

The original contributions presented in the study are included in the article/Supplementary Material, further inquiries can be directed to the corresponding author/s.

## Ethics Statement

All procedures followed during this investigation conform to the guide for the Care and Use of Laboratory Animals published by the US National Institutes of Health (NIH Publication No. 85–23, revised 1996) and the experimental protocol was approved by the Animal Welfare Committee of La Plata School of Medicine.

## Author Contributions

RD, EA, and AO: conceptualization. RD, CJ, and AO: general methodology. LDZ and EA: patch clamp. PB and RD: echocardiography. CV: electrocardiography. EP: histological analysis. RD and AO: statistical analysis. RD: writing—original draft preparation. AO and EA: writing—review and editing and funding acquisition. All authors contributed to the article and approved the submitted version.

## Funding

This study was supported by grants PICT 2019-1459 from Foncyt (AO) and PICT 2017-1567 from Foncyt (EA).

## Conflict of Interest

The authors declare that the research was conducted in the absence of any commercial or financial relationships that could be construed as a potential conflict of interest.

## Publisher's Note

All claims expressed in this article are solely those of the authors and do not necessarily represent those of their affiliated organizations, or those of the publisher, the editors and the reviewers. Any product that may be evaluated in this article, or claim that may be made by its manufacturer, is not guaranteed or endorsed by the publisher.
