# Supplementary material for: In vivo Overexpression of Electrogenic Sodium/Bicarbonate Cotransporter (NBCe1) by AAV9 Modifies the Cardiac Action Potential and the QT Interval in Mice
Source: Front Cardiovasc Med. 2022 Apr 25;9:862118. doi: 10.3389/fcvm.2022.862118 (PMC9082548; doi:10.3389/fcvm.2022.862118)
Supplement: Supplementary file 1 [file Data_Sheet_1.docx]

**In vivo overexpression of electrogenic sodium/bicarbonate cotransporter (NBCe1) by AAV9 modifies the cardiac action potential and the QT interval in mice**

**Di Mattía RA^1#^, Díaz-Zegarra LA^1#^, Valverde CA^1^, Blanco PG^2^, Jaquenod De Giusti C^1^, Portiansky EL^3^, Aiello EA^1^*, Orlowski A^1^*.**

**Supplementary material**

**Supplementary Table 1.** Individual values of each echocardiogram parameter for all the animals analysed before and after a month of injection with AAV9s. LVID: Left Ventricular Internal Dimension. IVST: Inter Ventricular Septal Thickness. LVPW: Left Ventricular Posterior Wall. LVMI: Left Ventricular Mass Index. FS: Fractional Shortening. HR: Heart Rate.


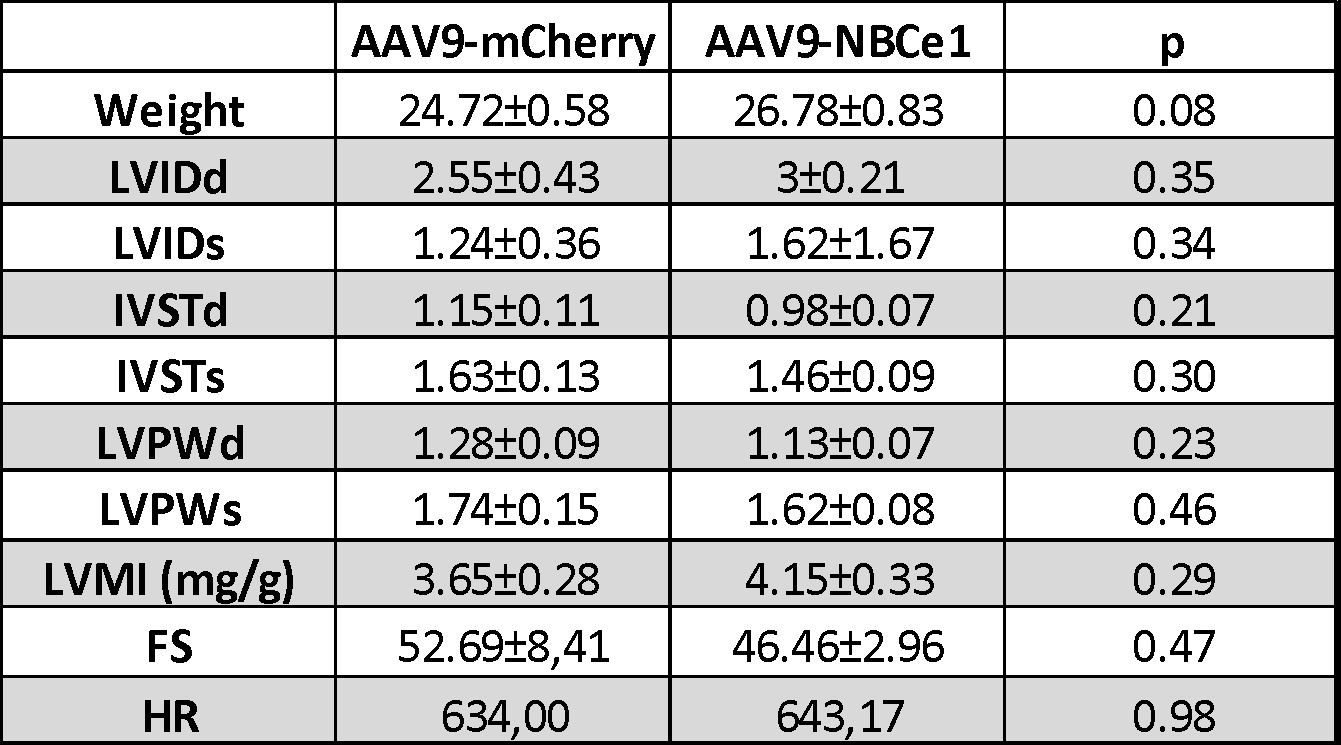


**Supplementary Table 2.** Mean expressed as ±SEM of each echocardiogram parameter after a month of injection with AAV9-mCherry and AAV9-NBCe1. p-values calculated with a Student t-test are included. AAV9-mCherry N=5, AAV9-NBCe1 N=6. LVID: Left Ventricular Internal Dimension. IVST: Inter Ventricular Septal Thickness. LVPW: Left Ventricular Posterior Wall. LVMI: Left Ventricular Mass Index. FS: Fractional Shortening. HR: Heart Rate.


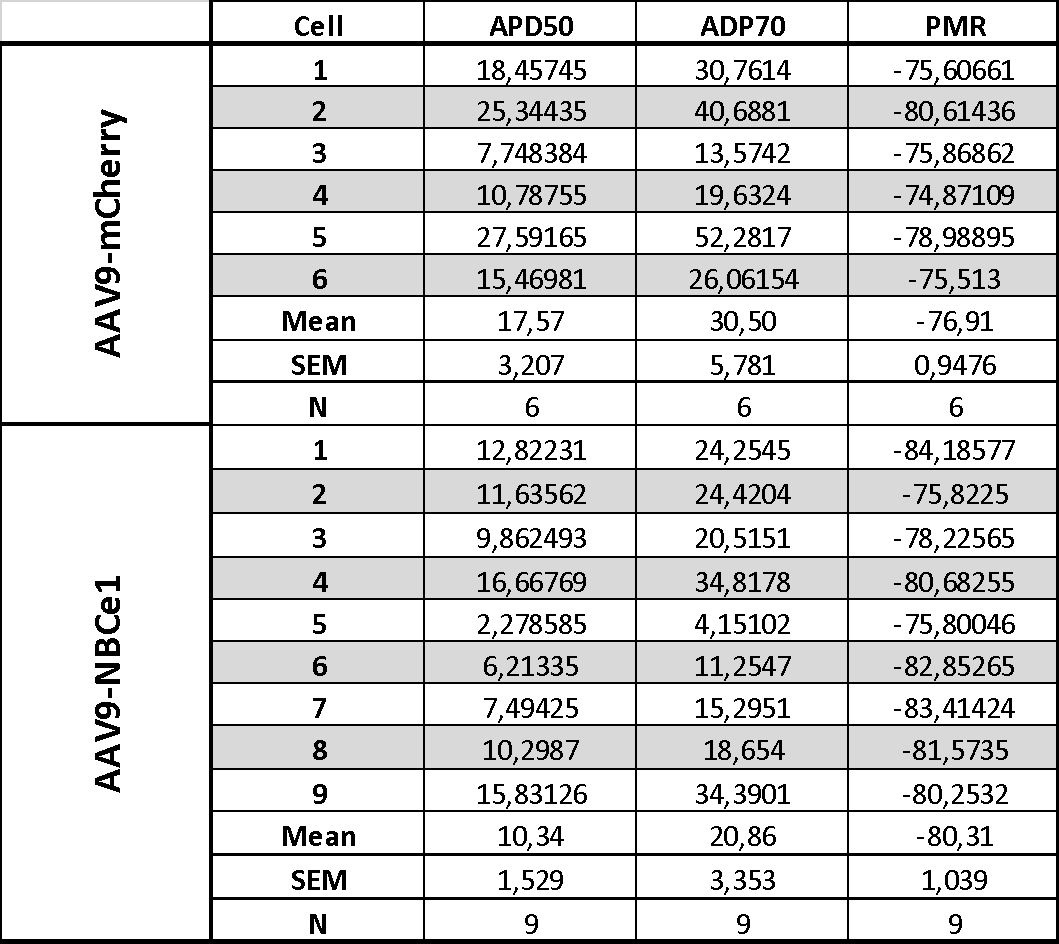


**Supplementary table 3:** Individual values of APD50, APD70 and PMR for each cell from AAV9-mCherry and AAV9-NBCe1 mice.


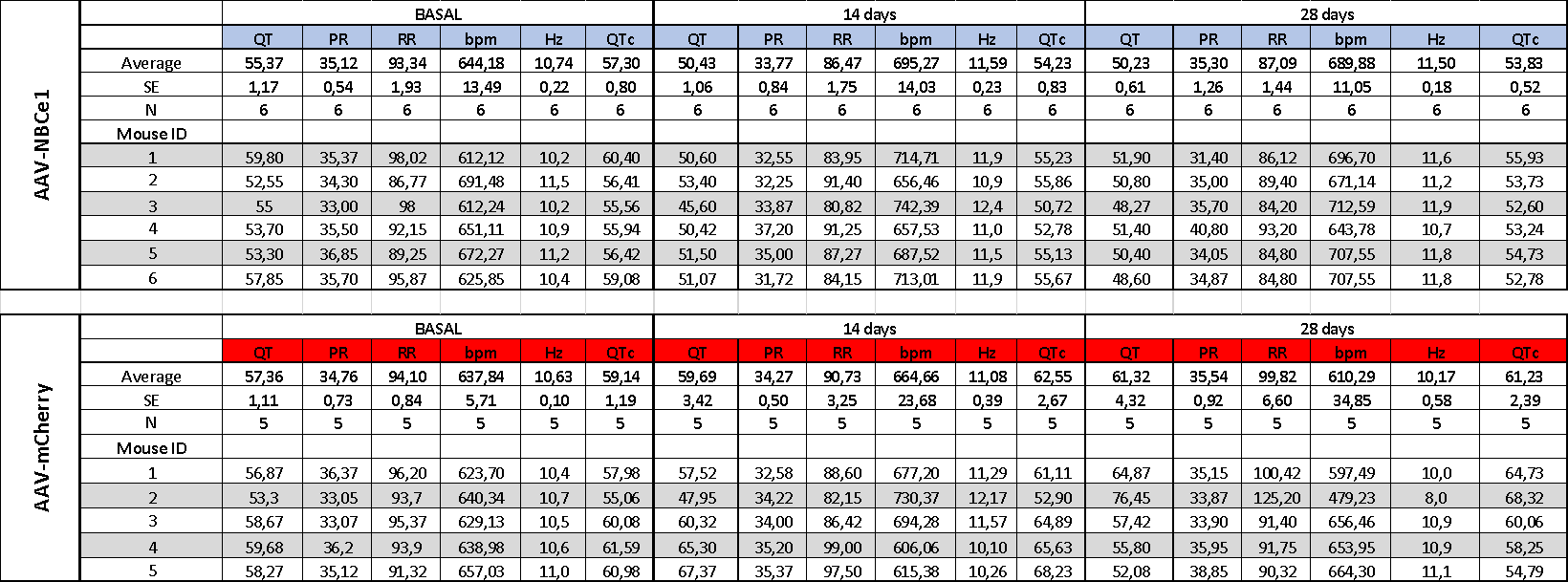


**Supplementary Table 4.** Individual values of electrocardiogram parameters for all the animals analysed before and after the injection with AAV9s. In each case, mean, SEM and number of animals were included. QTc was calculated with Mitchell formula QT/(RR/100)^1/2^. QT: QT interval. PR: PR interval. RR: RR interval. Bpm: beats per minute. Hz: hertz. QTc: corrected QT interval.


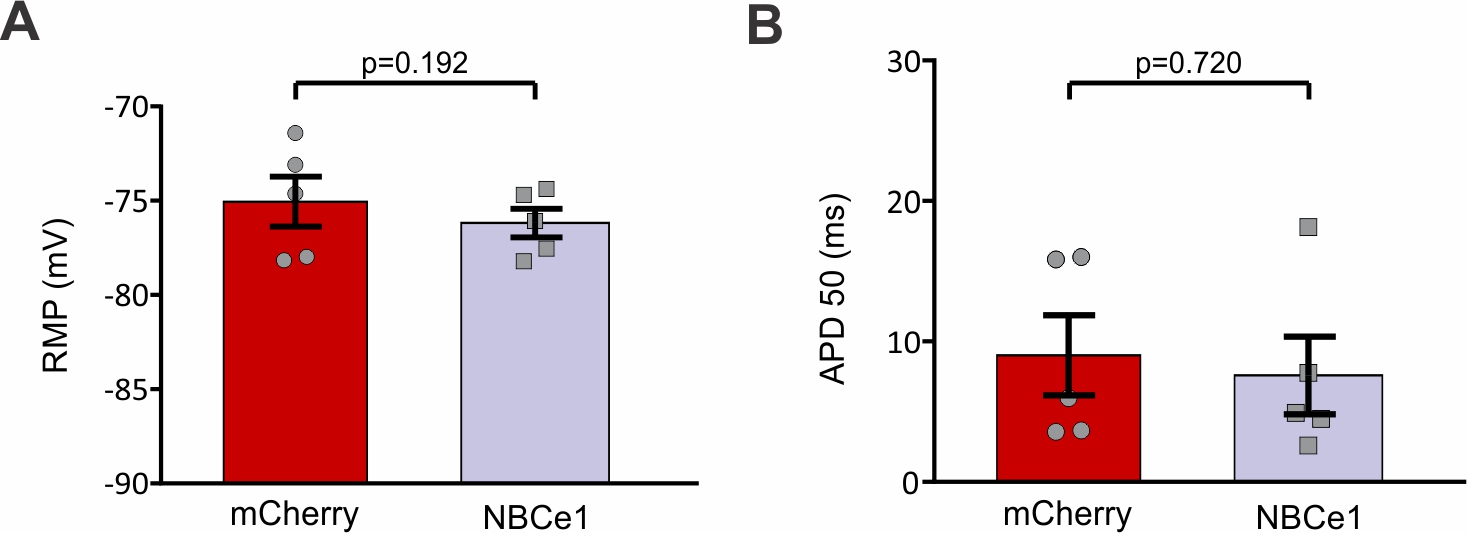


**Supplementary figure 1:** Resting membrane potential (RMP) (**A**) and action potential duration at 50% of repolarization (APD 50) (**B**) in a free bicarbonate buffer solution.


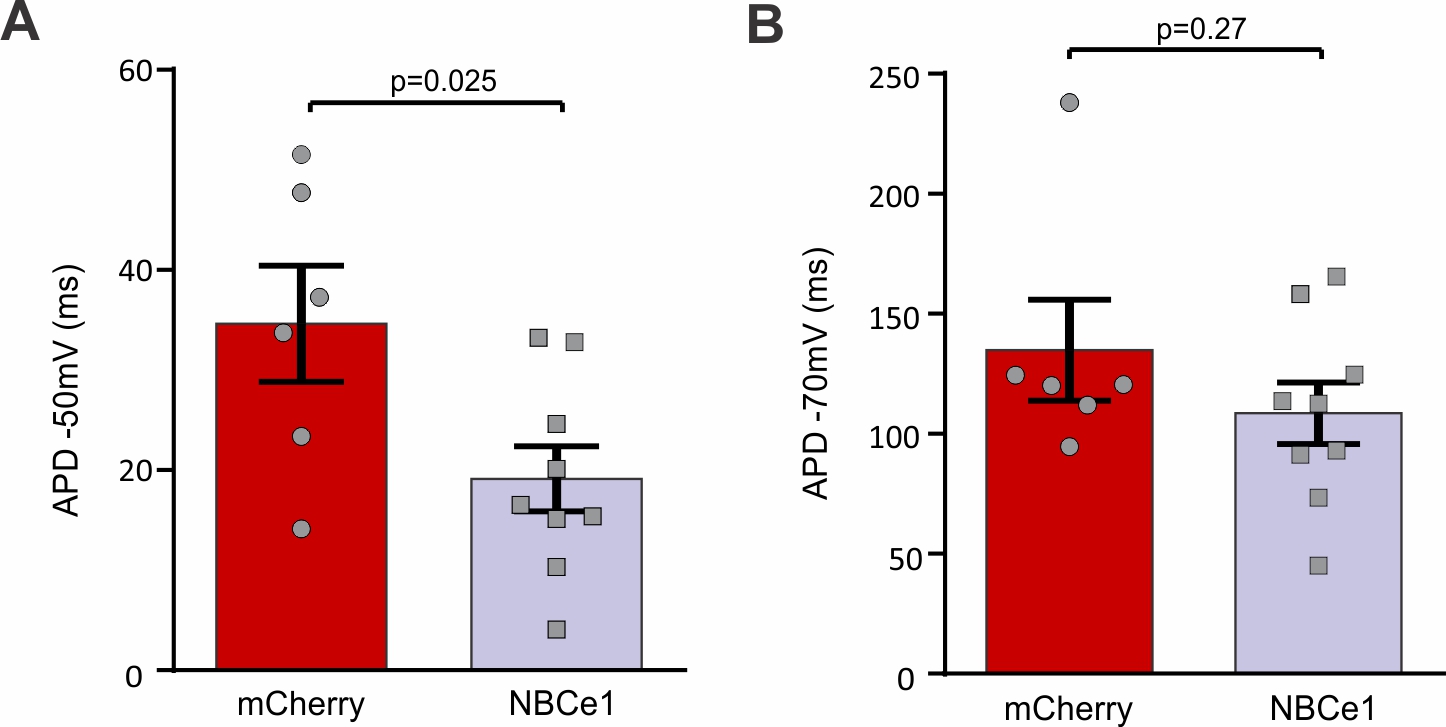


**Supplementary figure 2:** Action potential duration at -50mV (APD -50mV) (**A**) and -70mV (APD -70mV) (**B**).
